# Risk factors for “late-to-test” HIV diagnosis in Riverside County, California

**DOI:** 10.1097/MD.0000000000005021

**Published:** 2016-09-30

**Authors:** Aaron T. Gardner, Rachaline Napier, Brandon Brown

**Affiliations:** a Epidemiology & Program Evaluation Branch, Riverside University Health System Public Health; b University of California, Riverside, School of Medicine, Center for Healthy Communities, Riverside, CA.

**Keywords:** AIDS, HIV, HIV testing, late testing

## Abstract

Patients diagnosed late in the course of HIV infection are at an increased risk of negative health outcomes and are more likely to transmit HIV to others. Using the CDC's definition for AIDS, we analyzed case report data from persons diagnosed with AIDS within 12 months of an HIV diagnosis (“late-to-test”) in Riverside County, CA, between 2009 and 2014. Of 1385 HIV cases, 422 (30.5%) were late-to-test. Factors associated with late-to-test were: having no insurance (*P* *=* 0.005), being Hispanic (*P* *=* 0.002) and being between 45 and 64 years of age (*P* *<* 0.001). Females (*P* = 0.013) and those in the eastern region of Riverside County (*P* = 0.002) were less likely to be late-to-test. In the absence of universal HIV testing, interventions to decrease late testing are needed.

## 1 Introduction

As of December 31, 2014, Riverside County, CA, reported 5389 people living with HIV or AIDS. With an HIV prevalence >221 per 100,000 individuals, Riverside County is the sixth most HIV/AIDS-impacted county in California. Survival rates for HIV-infected persons have dramatically increased in the United States since the introduction of highly active antiretroviral therapy (HAART) in the mid-1990s. However, progress in obtaining early HIV diagnoses has been slow. The Centers for Disease Control and Prevention (CDC) estimate 1.2 million people live with HIV in the United States, and nearly 13% are unaware of their infection.^[1]^ Later stage HIV diagnosis (i.e., AIDS diagnosis within 12 months of seropositive determination) presents challenges to both individual treatment and public health prevention strategies.^[2,3]^ More often than not, late-stage patients do not get tested until symptoms have begun to appear.^[2,4,5]^ Previous studies indicate these individuals are often from marginalized groups, groups that do not recognize their risk potential, or both.^[2,6]^

Late-stage infection increases the risk of negative health outcomes, including premature death and substantially increases the overall treatment cost.^[3,7]^ Furthermore, late diagnosis increases the odds the unaware patient will transmit HIV.^[8]^ Nearly half (49%) of all new HIV infections are from those unaware of their HIV status.^[9]^ Nationally, 25% to 47% of individuals who present for HIV testing are late in the infection stage.^[10]^ Previous research has reported persons tested late in the course of their infection were more likely to be older, male, uninsured, and HIV-infected through heterosexual contact.^[2,6,11,12]^ Being foreign-born has been shown as a risk factor in some but not all studies.^[6]^ Those reporting injection drug use (IDUs), men who have sex with men (MSM), and heterosexual women are more likely to be diagnosed earlier in the course of their HIV infection.^[2,6,12]^ Inspired by similar work focused exclusively on AIDS cases in San Francisco, CA^[11]^, we examine the prevalence and risk factors for Riverside County residents who have an AIDS diagnosis within 12 months of their first HIV-positive test.

## 2 Methods

We used data from the Enhanced HIV/AIDS Reporting System (eHARS) maintained by the Office of AIDS, California Department of Public Health. The eHARS is a web-based application provided by the CDC to collect, manage, and report California's HIV/AIDS case surveillance data to the CDC. Data in eHARS come from both active and passive surveillance activities such as public health department review of medical records, laboratory reports, and health care provider reports. We examined the eHARS dataset including all persons who met the CDC surveillance case definition for HIV or AIDS (HIV infection, stage 3) within Riverside County, CA, from January 2009 to December 2014 and were reported to the Riverside University Health System through June 2015. Persons whose initial HIV diagnosis occurred within 12 months of their AIDS diagnosis were defined as late-to-test.

Data obtained from medical records included: age, gender at birth, race/ethnicity, HIV risk category, insurance status, foreign born, county region, date of HIV diagnosis, and date of AIDS diagnosis. Association between sociodemographic variables, sexual risk behaviors, and late testing was done using Pearson chi-square testing. Multivariate logistic regression determined independent predictors of late testing: all variables with a *P*-value of 0.2 or less from the initial chi-square test were entered into the regression model. Odds ratios (OR) and 95% confidence intervals (CIs) were calculated for the model. SPSS (Version 20.0; SPSS, Armonk, NY, 2011) was used for statistical analysis. As a public health practice, this evaluation of programmatic data is exempt from IRB review.

## 3 Results

**Figure 1:**
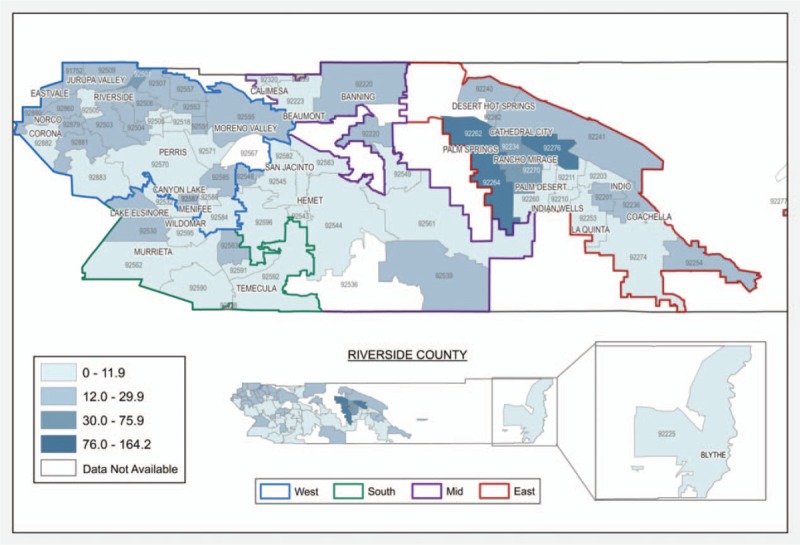
Late to test rates per 100,000 population by zip-code, Riverside County, 2009–2014.

The initial analysis included a total of 1424 Riverside County residents with an HIV diagnosis; 39 cases were excluded due to incomplete or erroneous HIV or AIDS diagnosis dates. We created a map of the late-to-test rates (Fig. 1) based on the remaining 1385 cases (97.3%) used for this analysis.

Of the 1385 cases, more than two-thirds were male (88%), born in the United States (72%), and either white or Hispanic (48% and 36%, respectively). Mean age was 39.5 years, with nearly half (44%) between the ages of 25 and 44 years. Insurance status for over one-third of participants was unknown (41%), whereas 22% and 25% had private or public insurance, respectively. Nearly half of the participants were from western Riverside County (40%) and the most common reported mode of HIV transmission was MSM (70%).

Of the total HIV cases, 30.5% (N = 422) were late-to-test (Table 1). Of those classified as late-to-test, 30.8% (N = 130) received their HIV and AIDS diagnoses at the same time. Bivariate analysis showed late-to-test was more frequent among Hispanics (OR 1.46, 95% CI 1.113–1.88), those between the ages of 45 to 64 years (OR 1.24, 95% CI 0.96–1.59), and in those born outside the United States (OR 1.60, 95% CI 1.13–2.27).

**Table 1:**
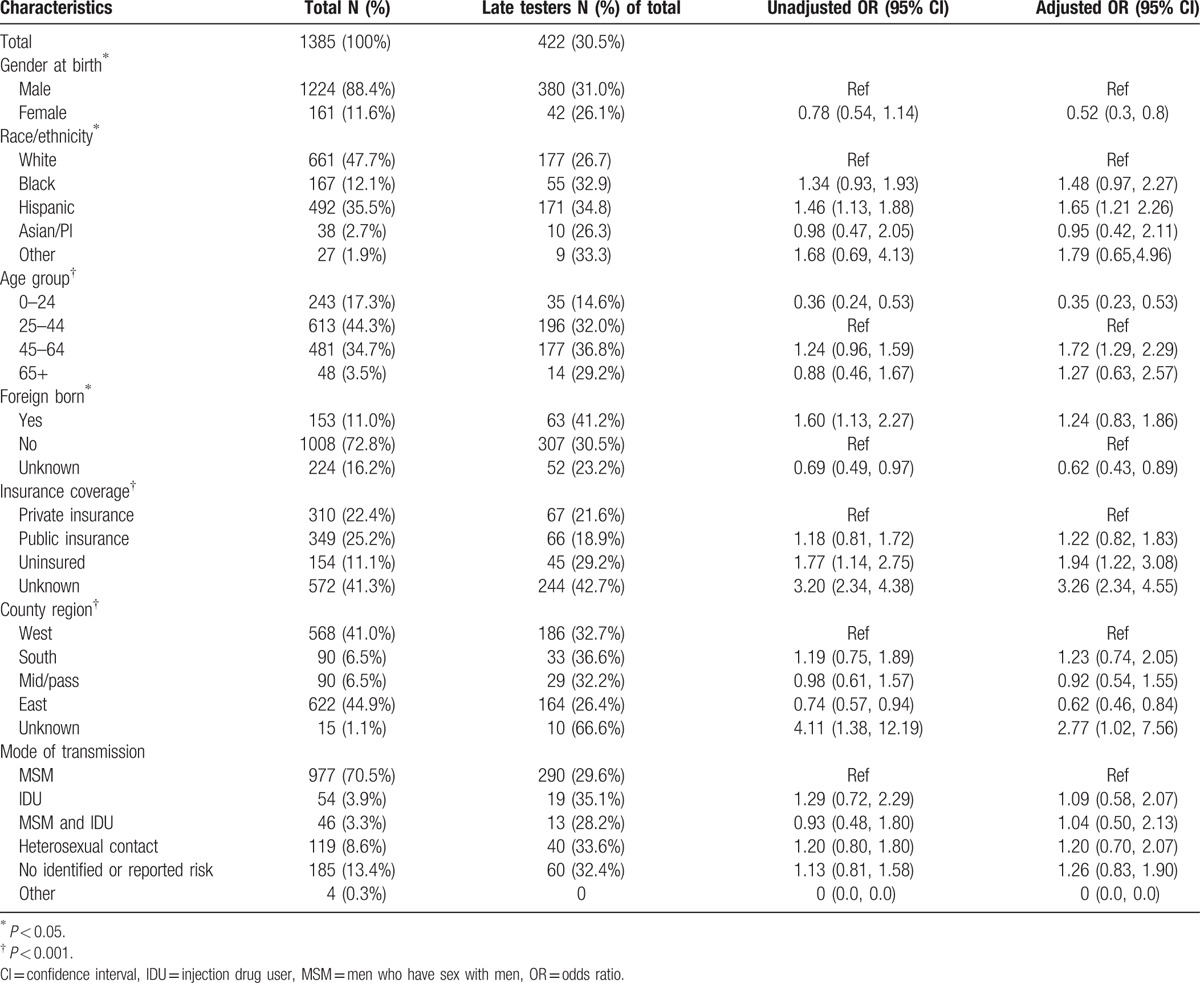
Characteristics and independent predictors of late-to-test persons with AIDS.

Those less likely to be late-to-test were under 25 years old (OR 0.36, 95% CI 0.24–0.53), and/or living in the eastern part of Riverside County (OR 0.74, 95% CI 0.57–0.94). Risk category (i.e., mode of transmission) had a limited effect on late testing (*P* *=* *0.77*). Factors independently associated with late-to-test were: having no insurance (OR 1.94, 95% CI 1.22–3.08), being Hispanic (OR 1.65, 95% CI 1.21–2.26), and being between the ages of 45–64 (OR 1.72, 95% CI 1.29–2.29). Females (OR 0.52, 95% CI 0.31–0.87) and those residing in the eastern region of the county (OR 0.62, 95% CI 0.46–0.84) were less likely to be late-to-test.

## 4 Discussion

The geographic disparities shown in Fig. 1 indicate one challenge for HIV-prevention efforts in the region. Although free confidential testing is available throughout Riverside County, >30% of new HIV diagnoses qualified as late-to-test (i.e., received an AIDS diagnosis within 12 months of a seropositive HIV result). With a clinical latency period of up to a decade from HIV infection to the onset of AIDS, earlier diagnosis is a critical component in preventing onward transmission of the virus and represents missed opportunities for treatment.^[10]^ Previous research suggests that the transmission rate for those unaware of their HIV status is 4 times higher (49%–66%) than via those who know their status.^[8,9]^

Similar to previous research discussed by Mukolo et al,^[6]^ our study found increased risk of late HIV testing among those who were between 45 and 64 years of age and the uninsured; we additionally found increased risk for late testing among Hispanics but not other racial/ethnic minorities. Our analysis also found reduced risk among women and those who live in the eastern part of the County. The reduced risk in the eastern part of the county is particularly interesting since >60% of all people living with HIV/AIDS in Riverside County live in this region.^[13]^ Because of this, it also has the most extensive HIV education and testing programs, which may contribute to the reduced risk of late testing.^[14]^

In contrast to other studies showing an increased risk of late-to-test for heterosexual males and decreased risk for men who have sex with men and injection drug users,^[2,6,12]^ we found no independent association between mode of HIV transmission and late-to-test.

Several limitations to this study need to be considered when interpreting the findings. The data used for this analysis were obtained from mandatory HIV and AIDS case reporting, which limits the data available for analysis. For example, although the education level was collected in eHARS, we were unable to include this in the analysis due to extensive missing data. Also, other important social determinants data, such as income or employment status, are not collected, nor are specific reasons why individuals might be late-to-test. Additionally, patients who test late but responded well to combination antiretroviral therapy, delaying or preventing onset of AIDS, were excluded from this analysis. Additionally, we were unable to detect those patients who dropped out of care for >1 year after initial HIV non-AIDS diagnosis.

Despite these limitations, our analysis provides important insights. Expansion of testing services, particularly into places with a high density of uninsured and/or Hispanic residents, should be pursued. Effort should be made to increase testing in those 30 years of age and older to account for the clinical latency period from HIV infection and onset of AIDS. Finally, routine testing is cost effective, recommended by the CDC, and should be promoted.^[15,16]^ Routine and universal testing has the capacity to significantly increase the time period between an HIV-positive diagnosis to an eventual AIDS diagnosis and can lead to longer healthier lives for those infected with HIV.

## Acknowledgments

The authors acknowledge Amy Hyong, MS, MPH, Wendy Hetherington, MPH, Riverside University Health System—Public Health, and Jo L. Gerrard, University of California, Riverside School of Medicine.
